# Developing a nanoparticle test for prostate cancer scoring

**DOI:** 10.1186/1479-5876-10-44

**Published:** 2012-03-09

**Authors:** Qun Huo, Sally A Litherland, Shannon Sullivan, Hillari Hallquist, David A Decker, Inoel Rivera-Ramirez

**Affiliations:** 1 NanoScience Technology Center and Department of Chemistry, University of Central Florida, 12424 Research Parkway Suite 400, Orlando, FL 32826, USA; 2 Florida Hospital Cancer Institute, 2501 North Orange Ave Suite 247, Orlando, FL 32804, USA

**Keywords:** Prostate cancer, Cancer aggressiveness, Biomarker, Nanoparticle, Molecular diagnostics

## Abstract

**Background:**

Over-diagnosis and treatment of prostate cancer has been a major problem in prostate cancer care and management. Currently the most relevant prognostic factor to predict a patient's risk of death due to prostate cancer is the Gleason score of the biopsied tissue samples. However, pathological analysis is subjective, and the Gleason score is only a qualitative estimate of the cancer malignancy. Molecular biomarkers and diagnostic tests that can accurately predict prostate tumor aggressiveness are rather limited.

**Method:**

We report here for the first time the development of a nanoparticle test that not only can distinguish prostate cancer from normal and benign conditions, but also has the potential to predict the aggressiveness of prostate cancer quantitatively. To conduct the test, a prostate tissue lysate sample is spiked into a blood serum or human IgG solution and the spiked sample is incubated with a citrate-protected gold nanoparticle solution. IgG is known to adsorb to citrate-protected gold nanoparticles to form a "protein corona" on the nanoparticle surface. From this study, we discovered that certain tumor-specific molecules can interact with IgG and change the adsorption behavior of IgG to the gold nanoparticles. This change is reflected in the nanoparticle size of the assay solution and detected by a dynamic light scattering technique. Assay data were analyzed by one-way ANOVA for multiple variant analysis, and using the Student *t-*test or nonparametric Mann-Whitney *U-*tests for pairwise analyses.

**Results:**

An inverse, quantitative correlation of the average nanoparticle size of the assay solution with tumor status and histological diagnostic grading was observed from the nanoparticle test. IgG solutions spiked with prostate tumor tissue exhibit significantly smaller nanoparticle size than the solutions spiked with normal and benign tissues. The higher grade the tumor is, the smaller the nanoparticle size is. The test particularly revealed large differences among the intermediate Grade 2 tumors, and suggested the need to treat them differently.

**Conclusion:**

Development of a new nanoparticle test may provide a quantitative measure of the prostate cancer aggressiveness. If validated in a larger study of patients with prostate cancer, this test could become a new diagnostic tool in conjunction with Gleason Score pathology diagnostics to better distinguish aggressive cancer from indolent tumor.

## Background

Prostate cancer (PCa) is the most common malignancy and the second leading cause of cancer death in American men. Using digital rectal examination (DRE) combined with the PSA (prostate specific antigen) test, most prostate cancer cases are now detected at an early stage. However, due to the lack of accurate tests to distinguish aggressive cancers from indolent tumors, prostate cancer is often over-treated. Post-surgery pathology analysis revealed that 30% of tumors removed by radical prostatectomy are deemed clinically insignificant and would not have required such invasive treatment [1]. Over-diagnosis and treatment of low-risk prostate cancer has serious and long-lasting side effect: as high as 70% of the patients who receive radical prostatectomy treatment will suffer a loss of sexual potency that cannot be remedied by drugs such as sildenafil citrate [2]. Currently the most relevant prognostic factor to predict a patient's risk of death due to PCa is the Gleason score of the biopsied tissue samples. However, pathological analysis is subjective, and the Gleason score is only a qualitative estimate of the cancer malignancy. Molecular biomarkers and diagnostic tests that can accurately predict the prostate tumor aggressiveness are rather limited.

**Figure 1:**
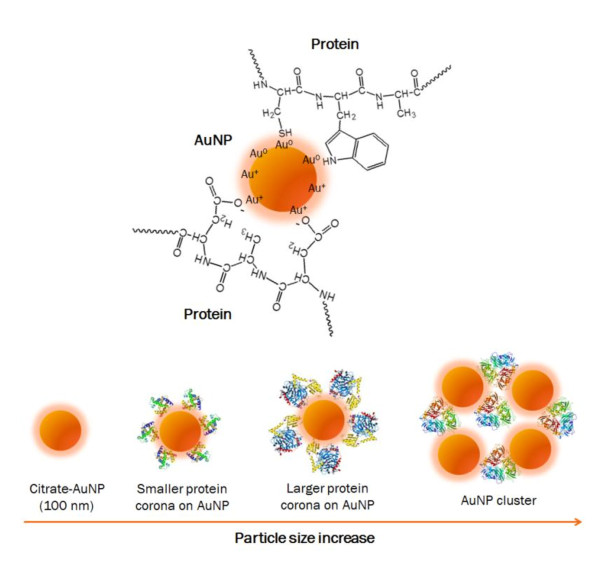
**A schematic illustration of "protein corona" formed on the surface of AuNPs upon protein adsorption**. The size of the protein corona varies depending on the type and the size of the proteins adsorbed to the AuNPs. As indicated, the adsorption of a protein complex or oligomer will cause larger nanoparticle size increase than an individual protein monomer. The protein-AuNP interaction may also cause AuNP cluster formation if multiple binding sites are present on a protein or protein complex. These differences can be readily detected and discerned according to the dynamic light scattering measurement.

We herein report on our work to develop a simple nanoparticle-serum protein adsorption test that not only can distinguish prostate cancer from normal and benign conditions, but also has the potential to predict the aggressiveness of prostate cancer quantitatively. This test is based on a new platform bioanalytical technology, nanoparticle-enabled dynamic light scattering assay (NanoDLSay) that we developed earlier [3-8]. NanoDLSay detects protein analytes by monitoring the nanoparticle size change upon specific binding or non-specific adsorption of target protein analytes to the gold nanoparticles (AuNPs). Citrate-protected AuNPs have a layer of negative charge on the surface because of the citrate ions. Proteins tend to adsorb to citrate-protected AuNPs through electrostatic interactions, Au-N and Au-S bonding to form a so-called "protein corona" (Figure 1) [9-11]. The size of the protein corona varies depending on the type and the size of the proteins adsorbed to the AuNPs. For example, as illustrated in the Figure 1, the adsorption of a protein complex or oligomer will cause larger nanoparticle size increase than an individual protein monomer. The protein-AuNP interaction may also cause AuNP cluster formation, if multiple binding sites are present in the proteins or protein complexes. These differences can be readily detected and discerned by dynamic light scattering (DLS) analysis, a technique used routinely for particle size measurement.

Blood serum contains a large number and a large quantity of various serum proteins. Recently, we proposed a serum-AuNP adsorption assay for cancer biomarker discovery based on a simple hypothesis that there may be some differences in the serum proteins adsorbed to the AuNPs between cancer and non-cancer samples [8]. To conduct the assay, a serum solution is simply mixed with a citrate-protected AuNP solution and the average particle size of the AuNP solution before and after sample incubation is measured by DLS. From our previous study conducted on mice models [8], we discovered that there is a significant difference in the serum-adsorbed AuNP size between mouse serum samples with and without prostate tumor. The average particle size of the assay solution is substantially smaller for mice carrying large tumor grown from orthotopically injected PC3 cells compared to healthy control mice and mice bearing smaller tumor grown from LnCaP cells [8]. However, from the previous study, the same difference was not observed from human serum samples with and without prostate cancer.

The biggest challenge for cancer biomarker discovery and early cancer detection is that at early stage, the amount of specific molecules that are released from the tumor to the blood is very small. In the mice model study we conducted previously [8], the relative tumor mass versus body weight of the PC3 and LnCaP mice was approximately 5% and 0.3%, respectively. These ratios would correspond to a tumor mass of 2.5 Kg and 150 g in a human patient with a body weight of 50 Kg. Such tumor sizes are far exceeding the tumor size from human patients with early stage cancer. The volume of a high grand human cancer is about 3.5-4.0 cc [12]. The only human setting with prostate cancer > 150 g would be the metastatic setting. It is therefore not surprising that the difference found from mice models was not observed from human serum samples.

In order to apply the AuNP adsorption test on human patient samples, we introduced a unique new experiment in this study to increase the amount of cancer-specific components by spiking the blood serum samples with primary tumor tissue extracts prior to the AuNP adsorption test. We hypothesize that when tumor-associated molecules are released to the blood, this may cause certain molecular changes to occur in the serum and such molecular changes are reflected in the AuNP-serum adsorption assay. By spiking a tumor tissue lysate directly to the blood, the concentration of tumor-associated molecules in the blood is synthetically increased, and as a result, molecular change of the serum similar to what occurs in *in vivo *may be more easily and clearly observed.

We have now indeed observed the very same difference from the human serum samples that we observed previously from mice models: the average particle size of human serum samples spiked with prostate tumor tissue is substantially smaller than the serum samples spiked with normal and benign prostate tissue lysates in the serum-AuNP adsorption test. It was discovered that this difference is caused by the interactions between tumor-specific molecules and the circulating immunoglobulin G (IgG) proteins in the blood serum. Such interactions changed the adsorption behavior of IgGs to the AuNPs, and subsequently, the average nanoparticle size of the assay solution. By spiking prostate tissue lysates into a pure human IgG solution, we observed the same particle size differences between normal, benign and prostate tumor tissues as observed from the blood serum samples. Furthermore, there is a significant, quantitative inverse correlation of the average nanoparticle size of the assay solution with tumor histological diagnostic grading. The current study is a preliminary in vitro report. If validated in a larger study of patients with prostate cancer, this serum protein (IgG)-AuNP adsorption assay can potentially become a new prognostic tool in conjunction with Gleason Score pathology diagnostics for better assessment of prostate cancer aggressiveness.

## Materials and methods

### Materials

**Figure 2:**
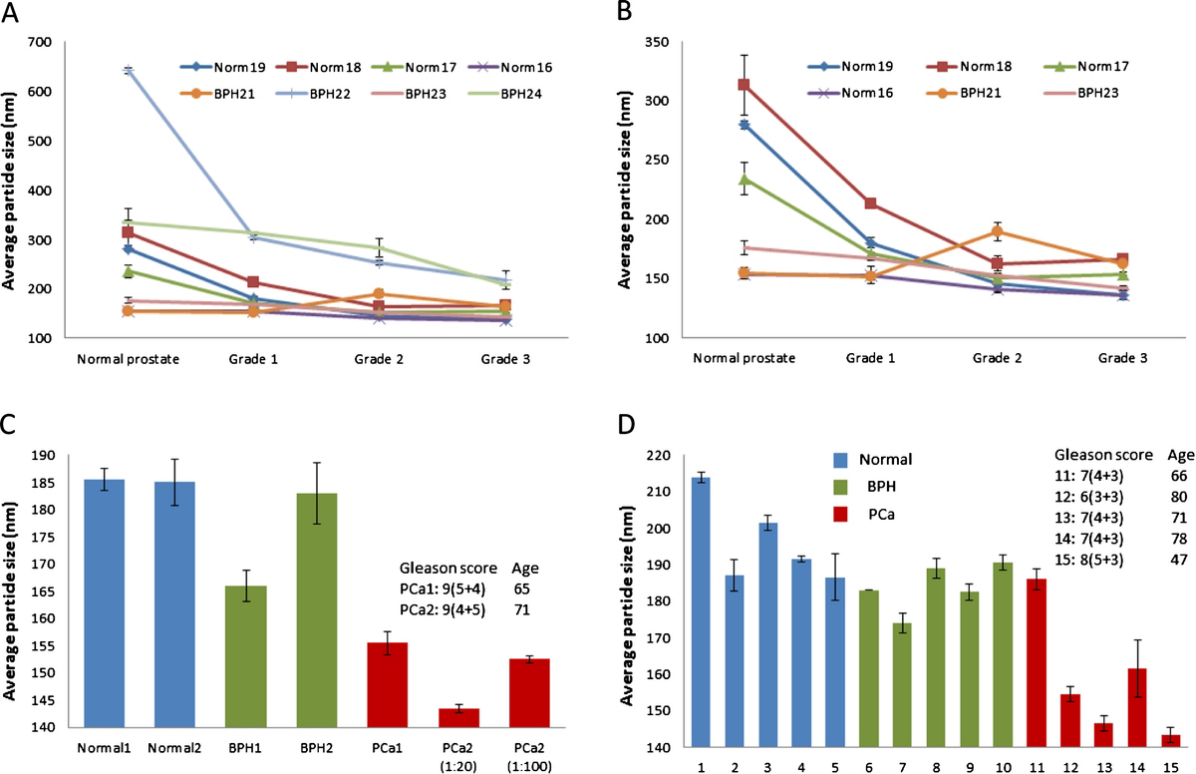
**The AuNP adsorption assay results of serum spiked with different prostate tissue lysates**. All samples were prepared by spiking 1 μL tissue lysate into 20 μL serum. All spiked samples were incubated at 4°C overnight before assay was conducted. Particle size was measured after 8 minutes of serum-AuNP incubation. A and B: the assay results of 8 serum (normal healthy donor = 4; BPH = 4) spiked with 4 prostate tissue lysates from normal, tissue with Grade 1, Grade 2, and Grade 3 prostate adenocarcinoma. The Gleason scores of the three tumor tissues are: 4(2 + 2), 5(2 + 3), and 9(5 + 4), respectively. A is the scatter-plot of all 32 samples and B is an expansion of A with 6 samples that have relatively smaller average particle sizes. Linear regression analysis of each sample set suggests that all but BPH21 sample mixes (R squared = 0.2061, p = 0.1382) had significant linear inverse correlations between the average particle size seen in the nanoparticle assay and the increasing tumor grade/staging, with goodness of fit R squared values ranging from 0.7406, p = 0.0003 for N17 to 0.9734, p < 0.0001 for BPH 23 sample sets. C and D: two sets of assay results of two different serum samples spiked with tissue lysates from normal healthy donors, BPH patients, and PCa donors. In the first set (C), PCa2 tissue lysate was spiked to the serum at two different ratios: 1:20 and 1:100 (tissue lysate:serum, v/v). Statistically significant differences were found in the assay results between the normal controls (mean 196.1 ± 11.3SD) and BPH samples (mean 183.8 ± 6.4SD; p = 0.0078, Student *t*-test), BPH and PCa samples (mean 158.4 ± 16.3SD; p = 0.0051, Mann-Whitney *U*-test), and normal and PCa samples (p < 0.0001, Student *t*-test).

**Figure 3:**
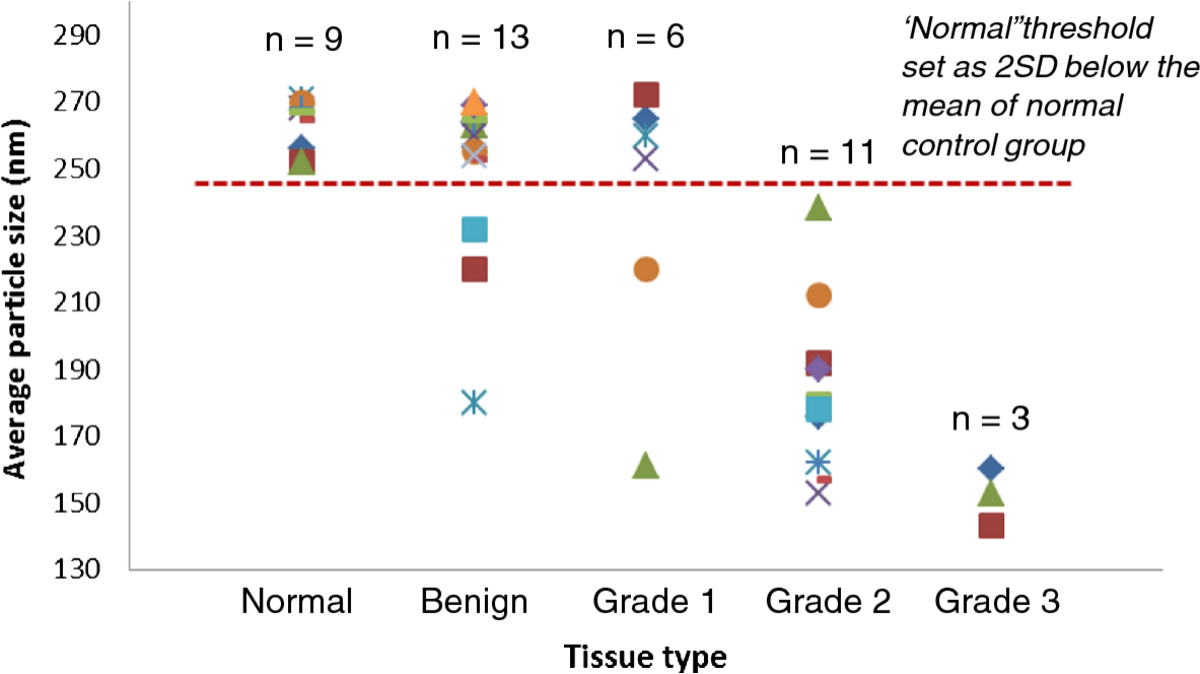
**The AuNP adsorption assay results of human IgG solution spiked with different prostate tissue lysates**. All samples were prepared by spiking 1 μL tissue lysate into 20 μL IgG solution. The concentration of IgG solution was 1 mg/mL in phosphate buffer (10 mM, pH 7.4). The prepared samples were incubated at r.t. for 30 min before assay was conducted. Particle size was measured after 3 minutes of human IgG-AuNP incubation. The 'normal range' threshold (red dotted line) was set as 2SD below the mean of the normal control group analyses.

Citrate-protected gold nanoparticle (AuNP) (15708-9) was purchased from Ted Pella Inc. (Redding, CA). The average diameter of the citrate AuNP is 100 nm and the concentration of the nanoparticle is 10 pM. Pure human IgG (ab91102) was purchased from Abcam (http://www.abcam.com). All human serum samples were purchased from Asterand Solutions (http://www.asterand.com). Tissue lysate samples were purchased from Protein Biotechnologies (http://www.proteinbiotechnologies.com). The protocol used for preparing the tissue lysates can be found from the website of Protein Biotechnologies and is also summarized in the Additional file 1 along with the clinical data of tissue samples. Data presented in each graph (Figures 2 and 3) were based on the assays of samples that were collected using the same method, stored under the same conditions, thawed, prepared, and tested at the same time under the exactly same assay conditions. All human tissue and serum samples used in this study are de-identified, archived specimens. The University of Central Florida IRB committee approved the use of these commercially acquired samples with exemption.

### Sample preparation and assay methods

To prepare tissue lysate-spiked serum samples or pure IgG solutions, 1 μL lysate at a total protein concentration of 1 mg/mL was mixed with 20 μL serum or IgG solution (concentration of 1 mg/mL in phosphate buffer, pH 7.4). The mixed solution was set at 4°C overnight for tissue lysate-spiked serum samples, and 30 minutes at room temperature for tissue lysate-spiked IgG samples before nanoparticle assay was conducted. To conduct the AuNP adsorption assay, 2 μL sample solution was mixed with 40 μL AuNP solution. The serum-AuNP solution was incubated for 8 min, and the IgG-AuNP solution was incubated for 3 min at room temperature before particle size was measured. All assays were conducted in duplicate and the error bars in each plot represent the standard error of the assay.

### Dynamic light scattering (DLS) analysis

Particle size analysis of the assay solutions was conducted using an automatic DLS instrument, *NDS1200*, from Nano Discovery Inc. (http://www.nanodiscoveryinc.com). This system is equipped with a 12-sample holder carousel to allow automatic measurement of 12 samples within 5-6 minutes. The measurement error for the pure AuNP solution with an average diameter of 100 nm is ± 2 nm. All DLS measurements were conducted at an ambient temperature of 25°C. The polydispersity index of the pure citrate-protected AuNPs and all AuNP assay solutions (i.e., the mixed solution of AuNPs and samples) falls in the range of 0.3-0.5. There is no significant difference between pure AuNP and AuNP assay solution.

### Statistical analysis

Where possible, data were analyzed by one-way ANOVA for multiple variant analysis, and using Student *t-*test or nonparametric Mann-Whitney *U *-tests for pairwise analyses. The results of these analyses are included in the Additional file 1 with appropriate *p *values and statistical parameters (*n*, mean, SD and/or SEM). Linear regression analysis for data presented in Figure 2 was done with significance determined by comparing slope deviation from zero and the R squared value for fit on each of the assay sets presented.

## Results and discussions

We first conducted the serum-AuNP adsorption assay on a series of blood serum samples spiked with different types of prostate tissue lysates (Figure 2). In a first set of experiment, we tested 8 male serum samples (4 from normal donors and 4 from patients with abnormal but non-cancerous benign prostate hyperplasia, BPH) spiked with 4 different prostate tissue lysates that were from normal healthy control, tissue with Grade 1 (Gleason Score 4(2 + 2)), Grade 2 (Gleason Score 5(2 + 3)), and Grade 3 (Gleason Score 9(5 + 4)) prostate adenocarcinoma (Figure 2A and 2B, plot B is an expansion of plot A). All tissue lysates were prepared in the same buffer (a modified RIPA buffer) using exactly the same protocol and all tissue lysates have the same total protein concentration of 1 mg/mL. Among the 8 sets of serum samples, 7 sets exhibited a clear trend of decreased average particle size when the serum was spiked with prostate tumor tissue lysates. The average particle size is inversely related to the grade of the tumor. We tested additional sets of normal and tumor tissue lysates-spiked serum samples (including data presented here, total approximately 100 samples made from the combination of 10 serum samples spiked with 10 different tissue lysates), and the tests all showed the same trend of nanoparticle size reduction. BPH21 is the only exception observed throughout the whole study so far. Linear regression analyses suggest that all but BPH21 sample mixes (R squared = 0.2061, p = 0.1382) had significant linear inverse correlations between the average particle size seen in the nanoparticle assay and the increasing tumor grade/staging, with goodness of fit R squared values ranging from 0.7406, p = 0.0003 for N17 to 0.9734, p < 0.0001 for BPH 23 sample sets.

In a second set of experiment, we tested two sets of normal serum samples spiked with normal, BPH, and PCa tissue lysates, respectively. In the first set of samples (Figure 2C), the two PCa tissue lysates are both from Grade 3 tumor: PCa1 has a Gleason Score of (5 + 4) and PCa2 has a Gleason Score of (4 + 5). Again, the spiking of PCa tissue lysates to the serum led to a much smaller average particle size of the assay solution. Between the two BPH tissue lysates, one behaved like the normal tissue, and another one caused the particle size decrease of the assay solution; however, the decrease is smaller than the PCa tissue lysates. There is also a difference between the two PCa tissue samples: PCa2 caused more substantial particle size decrease than PCa1, even though PCa1 has a Gleason score of (5 + 4) while PCa2 has a Gleason score of (4 + 5). In contrast to the pathological analysis, the AuNP adsorption assay we conducted here suggests that PCa2 is more aggressive than PCa1. We also observed a concentration-dependent effect: PCa2 tissue lysate was spiked into the same serum in 1:20 and 1:100 (lysate:serum, v/v) ratio, respectively. With an increased amount of tissue lysate spiked into the serum, the particle size decreasing effect caused by the tumor tissue lysate is more dramatic.

The second set of samples showed very similar results (Figure 2D): the BPH tissue lysate-spiked samples showed slight nanoparticle size reduction compared to the normal samples, while 4 out of 5 tumor tissue lysate-spiked samples showed substantial nanoparticle size reduction compared to the normal tissue lysates. The most aggressive tumor among the five samples, #15 from a donor of age 47 with a Gleason score of 8, showed the largest nanoparticle size reduction. On the other hand, sample #11, a tumor with a Gleason score of 7, exhibited a similar behavior as a normal tissue sample. Even with the small number of samples tested in this study, a marked discrepancy can already be seen between the new test results and the pathology reports. Overall, statistically significant differences were found in the assay results between the normal controls (mean 196.1 ± 11.3SD), BPH (mean 183.8 ± 6.4SD), and PCa samples (mean 158.4 ± 16.3SD). The *p *value for the group pairs, normal-PCa, BPH-PCa, and normal-BPH, is < 0.0001, 0.0051, and 0.0078, respectively.

We want to emphasize that the observed difference between prostate cancer and non-cancer samples is not due to the buffer effect, because all tissue lysates samples were prepared using exactly the same buffer and the same procedure. We also tested serum samples spiked with the pure buffer solution (a modified RIPA buffer) that was used to prepare the tissue lysates, and no effect from the buffer on the serum-AuNP adsorption assay was observed at all.

Results obtained from our previous study on mice models and the current studies on human serum samples suggest the following: (1) When prostate tumor develops, certain chemicals and biomolecules are released from the tumor site to the blood and these chemicals/biomolecules interact with certain proteins in the blood, changing the serum protein adsorption to the AuNPs. (2) The more aggressive the prostate tumor is, the more substantial the serum molecular change is. In the search of a molecular mechanism to explain the observed results, a critical question to be answered is: what is the most relevant protein in the blood serum that has been changed by the tumor tissue chemicals/biomolecules?

Based on our own and others' studies [8,9], we suggested that in the serum-AuNP adsorption assay, the major components of the protein corona formed on the AuNPs are abundant serum proteins. Circulating immunoglobulin G (IgG) is one of the most abundant blood serum proteins, with a typical concentration in the range of 5-15 mg/mL. IgG is known to have strong affinity towards citrate-protected AuNPs, a property that has been used for decades as a general method to prepare AuNP immunoprobes through a simple adsorption process [13]. If IgG is indeed a major component in the protein corona adsorbed to the AuNPs, the observed difference between cancer and non-cancer samples could then have been due to the unique interactions between tumor-specific molecules and IgG.

To test this hypothesis, we conducted the same AuNP adsorption assay on pure human IgG solution spiked with 42 prostate tissue lysates. Remarkably, we observed the same particle size differences between tumor, benign and normal tissue-spiked IgG solution (Figure 3, *n *is the number of tissue samples tested). Furthermore, the average particle size of the assay solution is inversely related to the tumor grade. The normal and the most aggressive Grade 3 tumor can be clearly differentiated without any overlap. Most benign and Grade 1 tumor tissues gave similar results as normal tissues, but with two samples resembling a more aggressive tumor profile. The assay results of 11 Grade 2 tumor tissues extend over a wide range, reflecting exactly the ambiguous aggressiveness of the Grade 2 tumor. According to the pathological reports, all these Grade 2 tumors are the same or similar; however, based on the nanoparticle test, there are substantial differences between these intermediate grade tumors, and should be treated differently. If the 'normal range' threshold is set as 2SD below the mean of the normal control group (red dotted line), the Grade 2 and 3 tumors are detected with 100% sensitivity from the nanoparticle test. However, most low-grade prostate tumors are slow-growing tumors and may not need to be treated with the radical prostatectomy surgery. To determine the best cut-off value/range for treatment selection, a more extensive clinical study based on a larger data set needs to be conducted.

**Figure 4:**
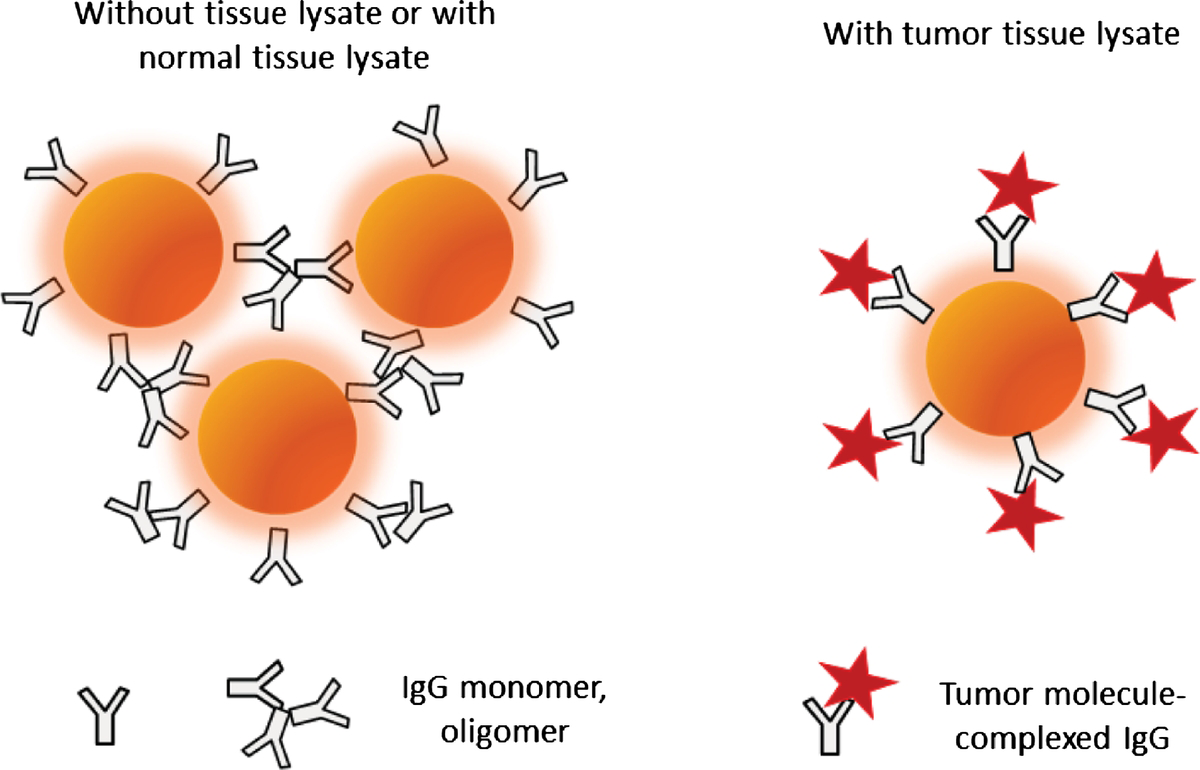
**A mechanistic model to explain the observed difference between normal and prostate cancer samples**. Human IgG, either as a monomer or oligomer, causes AuNP cluster formation when adsorbed to the AuNPs. When mixing IgG solution or serum with tumor tissue lysates, the specific binding of tumor-associated molecules with IgG inhibits the crosslinking of the AuNPs, leading to a decreased average nanoparticle size of the assay solution.

There are two hypotheses that may explain the nanoparticle size reduction caused by the spiking of tumor tissue lysates into serum or pure IgG solution. One is that the molecules released from the tumor tissue compete with the serum proteins/molecules to bind with AuNPs. For some reasons, the tumor tissue-associated molecules adsorbed to the AuNPs are smaller than the serum proteins, and also smaller than the normal tissue-associated molecules, leading to the observed nanoparticle size reduction of tumor tissue-spiked serum or human IgG solution. However, this hypothesis is not supported by the results of a control experiment: we conducted the AuNP adsorption assay directly on tumor tissue and normal tissue lysate solutions, and found that the tumor tissue lysates generally led to a slightly larger nanoparticle size increase than the normal tissue lysates (by about 10 nm). A second hypothesis is the more plausible reason: certain tumor tissue-specific molecules interact with serum IgG to form complexes, and this interaction changed the IgG adsorption to AuNPs. A model as illustrated in Figure 4 is proposed to explain the observed difference between cancer and non-cancer samples. IgG, either as a monomer or oligomer, causes AuNP cluster formation when adsorbed to the AuNPs. When mixing IgG solution or serum with tumor tissue lysates, the specific binding of tumor-specific molecules with IgG inhibits the crosslinking of the AuNPs, leading to a decreased average nanoparticle size of the assay solution.

This mechanism suggests that serum IgG, by interacting with tumor-associated, enhanced or altered molecules, may be providing a natural immune defense against prostate cancer spreading in the blood. If this natural defense system is exhausted, cancer metastasis can begin. This model, very interestingly, echoes well with the findings on the association between cancer and the immune system/functions [14-16].

The above model also implicates that the best serum biomarker for early detection of aggressive prostate cancer may be found from the complexes with serum IgG, not as individual molecules in the blood. If the tumor-specific molecules are interacting with circulating IgGs at a typical antibody-antigen binding constant of 10^10 ^M^-1^, a simple calculation (assuming the serum IgG concentration is at an average value of 10 mg/mL) reveals that the concentration of IgG-complexed tumor biomarkers would be about 600,000 times of the free biomarker molecules in the blood. This means at early stage of cancer development, almost all cancer biomarker molecules released from the tumor site to the blood are complexed to the serum IgGs. Current bioassays are set almost exclusively to detect target protein analytes in individual molecular forms, without considering the complex behavior of a protein analyte. This is perhaps one reason to explain why there are so many inconsistencies in cancer biomarker research.

## Conclusions

In summary, we reported here the development of a simple nanoparticle assay that may be used for quantitative assessment of the prostate tumor aggressiveness. The significant inverse correlation of the average nanoparticle size of the assay solution with tumor histological diagnostic grading suggests that the nanoparticle assay could potentially provide a more accurate diagnostic tool to assess the tumor aggressiveness than the current diagnostic practices. Based on the results reported here and our previously reported studies on mice models, a large scale clinical study to validate the new nanoparticle test for prostate cancer diagnosis is justified. If successfully validated, the reported new test can bring an immediate solution to the long-standing over-treatment problem in prostate cancer care. Current assay still requires the use of biopsied tissue samples. It would be ideal if aggressive prostate cancer can be detected early using only blood samples. We are conducting further studies at the present to identify the specific tumor molecules that are complexed with the serum IgGs. We expect sensitive serum biomarkers for early detection of aggressive prostate cancer to be discovered from these studies.

## Competing interests

QH is an owner and officer of Nano Discovery Inc., a company that has licensed the NanoDLSay™ technology from University of Central Florida for commercialization. Other authors declare no competing financial interest.

## Authors' contributions

QH developed the conceptual framework of the study, designed the experiments, conducted studies, analyzed data and prepared the paper. SAL participated in the design of the experiment, explanation of the experimental data, conducted the statistical analysis of the data, and prepared the paper. SS and HH assisted QH to conduct the experiments. DAD and IR provided clinical insights to the study and data explanation, and participated in the preparation of the paper. All authors read and approved the final manuscript.

## Authors' information

QH (Ph.D.) is an Associate Professor in the NanoScience Technology Center with a joint appointment in the Department of Chemistry and Burnett School of Biomedical Science at University of Central Florida. She is an established expert in nanobiotechnology research and has published more than 30 papers in the last few years related to the development of gold nanoparticles for biomedical applications, especially for cancer biomarker research. Her publications are widely cited by the scientific community. SAL (Ph.D.) is the head of the Florida Hospital Translational Research Division. She is a seasoned research scientist with over 36 years of laboratory experience, and over 13 years as laboratory head/director, 18 years of experience working with clinical trials and human blood samples. Her research expertise includes inflammatory myeloid cell defects, human monocyte analyses, and immunological involvement in pathological conditions as well as extensive experience in protein, antigen, and immunohistochemical analysis methods. DAD (MD) is the Director of the Florida Hospital Cancer Institute. He is an internationally known expert oncologist with extensive experience both in clinical practice and clinical research. IR (MD) is the Lead Urologist in Winter Park Urology Associates. He is an internationally known expert in prostate cancer and robotic urologic surgery with extensive experience both in clinical practice and clinical research.

## Supplementary Material

Additional file 1 **The protocol used for tissue lysate preparation, the clinical data of the tissue samples, and the statistical analysis results of the assay data are included in the Supplementary Information**. Click here for file
