# Supplementary material for: Developing a nanoparticle test for prostate cancer scoring
Source: J Transl Med. 2012 Mar 9;10:44. doi: 10.1186/1479-5876-10-44 (PMC3337274; doi:10.1186/1479-5876-10-44)
Supplement: Additional file 1 — The protocol used for tissue lysate preparation, the clinical data of the tissue samples, and the statistical analysis results of the assay data are included in the Supplementary Information. [file 1479-5876-10-44-S1.DOCX]

Supplementary Information

**Developing a Nanoparticle Test for Prostate Cancer Scoring**

Qun Huo^1*^, Sally A. Litherland^2^, Shannon Sullivan^1^, Hillari Hallquist^1^, David A. Decker^2^, Inoel Rivera-Ramirez^2^

^1^NanoScience Technology Center and Department of Chemistry, University of Central Florida, 12424 Research Parkway Suite 400, Orlando, FL 32826

^2^Florida Hospital Cancer Institute, 2501 North Orange Ave Suite 247, Orlando, FL 32804

*Correspondence: Professor Q. Huo, Email: Qun.Huo@ucf.edu

*Protocols for tissue lysate preparation (as provided by Protein Biotechnologies):*

The tissue (frozen immediately upon surgery) is homogenized in the RIPA buffer to obtain the soluble proteins, and centrifuged to clarify. All samples used in the current study are the clear solution portion of the lysates. The composition of the modified RIPA buffer is the following: PBS (pH 7.4), 1 mM EDTA, 0.25% Na deoxycholate, 1 mM Na_3_VO_4_, 1 mM NaF, 0.1% SDS, 1 mM PMSF, 1 µg/mL aprotinin, 1 µg/mL pepstatin-A, and 1 µg/mL leupeptin. All clear lysate solutions are adjusted using the same buffer into a total protein concentration of 1 mg/mL.

Clinical Information of Prostate Tissue Samples (as provided by Protein Biotechnologies)

| Human Prostate Tissue Lysates | | | | | | | |
| --- | --- | --- | --- | --- | --- | --- | --- |
| **Catalog Number** | **Diagnosis** | **Grade** | **Gleason Score** | **Stage** | **TNM** | **Sex** | **Age** |
| [T3-001](http://www.proteinbiotechnologies.com/products/prostate/t3_001_human_prostate_tissue_lysate.html) | Prostatic adenoma | n/a | n/a | n/a | n/a | M | 74 |
| [T3-002](http://www.proteinbiotechnologies.com/products/prostate/t3_002_human_prostate_tissue_lysate.html) | Prostatic hypertrophy | n/a | n/a | n/a | n/a | M | 79 |
| [T3-003](http://www.proteinbiotechnologies.com/products/prostate/t3_003_human_prostate_tissue_lysate.html) | Benign prostatic fibrosis | n/a | n/a | n/a | n/a | M | 72 |
| [T3-004](http://www.proteinbiotechnologies.com/products/prostate/t3_004_human_prostate_tissue_lysate.html) | Prostatic fibroadenoma | n/a | n/a | n/a | n/a | M | 40 |
| [T3-005](http://www.proteinbiotechnologies.com/products/prostate/t3_005_human_prostate_tissue_lysate.html) | Normal prostate | n/a | n/a | n/a | n/a | M | 40 |
| [T3-006](http://www.proteinbiotechnologies.com/products/prostate/t3_006_human_prostate_tissue_lysate.html) | Normal prostate | n/a | n/a | n/a | n/a | M | 24 |
| [T3-007](http://www.proteinbiotechnologies.com/products/prostate/t3_007_human_prostate_tissue_lysate.html) | Prostatic hyperplasia | n/a | n/a | n/a | n/a | M | 65 |
| [T3-008](http://www.proteinbiotechnologies.com/products/prostate/t3_008_human_prostate_tissue_lysate.html) | Prostatic fibroma | n/a | n/a | n/a | n/a | M | 68 |
| [T3-009](http://www.proteinbiotechnologies.com/products/prostate/t3_009_human_prostate_tissue_lysate.html) | Prostatic hypertrophy | n/a | n/a | n/a | n/a | M | 81 |
| [T3-010](http://www.proteinbiotechnologies.com/products/prostate/t3_010_human_prostate_tissue_lysate.html) | Normal prostate | n/a | n/a | n/a | n/a | M | 24 |
| [T3-011](http://www.proteinbiotechnologies.com/products/prostate/t3_011_human_prostate_tissue_lysate.html) | Prostatic hypertrophy | n/a | n/a | n/a | n/a | M | 70 |
| [T3-012](http://www.proteinbiotechnologies.com/products/prostate/t3_012_human_prostate_tissue_lysate.html) | Prostatic hypertrophy | n/a | n/a | n/a | n/a | M | 84 |
| [T3-013](http://www.proteinbiotechnologies.com/products/prostate/t3_013_human_prostate_tissue_lysate.html) | Prostatic fibroma | n/a | n/a | n/a | n/a | M | 69 |
| [T3-014](http://www.proteinbiotechnologies.com/products/prostate/t3_014_human_prostate_tissue_lysate.html) | Prostatic hypertrophy | n/a | n/a | n/a | n/a | M | 85 |
| [T3-015](http://www.proteinbiotechnologies.com/products/prostate/t3_015_human_prostate_tissue_lysate.html) | Prostatic fibroma | n/a | n/a | n/a | n/a | M | 69 |
| [T3-016](http://www.proteinbiotechnologies.com/products/prostate/t3_016_human_prostate_tissue_lysate.html) | Normal prostate | n/a | n/a | n/a | n/a | M | 50 |
| [T3-017](http://www.proteinbiotechnologies.com/products/prostate/t3_017_human_prostate_tissue_lysate.html) | Normal prostate | n/a | n/a | n/a | n/a | M | 65 |
| [T3-018](http://www.proteinbiotechnologies.com/products/prostate/t3_018_human_prostate_tissue_lysate.html) | Prostatic hypertrophy | n/a | n/a | n/a | n/a | M | 80 |
| [T3-019](http://www.proteinbiotechnologies.com/products/prostate/t3_019_human_prostate_tissue_lysate.html) | Normal prostate | n/a | n/a | n/a | n/a | M | 37 |
| [T3-020](http://www.proteinbiotechnologies.com/products/prostate/t3_020_human_prostate_tissue_lysate.html) | Normal prostate | n/a | n/a | n/a | n/a | M | 61 |
| [T3-021](http://www.proteinbiotechnologies.com/products/prostate/t3_021_human_prostate_tissue_lysate.html) | Normal prostate | n/a | n/a | n/a | n/a | M | 32 |
| [T3-022](http://www.proteinbiotechnologies.com/products/prostate/t3_022_human_prostate_tissue_lysate.html) | Normal prostate | n/a | n/a | n/a | n/a | M | 40 |
| [T3-023](http://www.proteinbiotechnologies.com/products/prostate/t3_023_human_prostate_tissue_lysate.html) | Prostatic Adenocarcinoma | 1 | 5(3+2) | n/a | n/a | M | 79 |
| [T3-024](http://www.proteinbiotechnologies.com/products/prostate/t3_024_human_prostate_tissue_lysate.html) | Prostatic Adenocarcinoma | 2 | 7(4+3) | n/a | n/a | M | 77 |
| [T3-025](http://www.proteinbiotechnologies.com/products/prostate/t3_025_human_prostate_tissue_lysate.html) | Prostatic Adenocarcinoma | 3 | 9(5+4) | III | T3aNxMx | M | 65 |
| [T3-026](http://www.proteinbiotechnologies.com/products/prostate/t3_026_human_prostate_tissue_lysate.html) | Prostatic Adenocarcinoma | 2 | 7(4+3) | n/a | n/a | M | 66 |
| [T3-027](http://www.proteinbiotechnologies.com/products/prostate/t3_027_human_prostate_tissue_lysate.html) | Prostatic Adenocarcinoma | 1 | 4(2+2) | n/a | n/a | M | 63 |
| [T3-028](http://www.proteinbiotechnologies.com/products/prostate/t3_028_human_prostate_tissue_lysate.html) | Prostatic Adenocarcinoma | 1 | 6(3+3) | n/a | n/a | M | 76 |
| [T3-029](http://www.proteinbiotechnologies.com/products/prostate/t3_029_human_prostate_tissue_lysate.html) | Prostatic Adenocarcinoma | 2 | 7(4+3) | n/a | n/a | M | 66 |
| [T3-030](http://www.proteinbiotechnologies.com/products/prostate/t3_030_human_prostate_tissue_lysate.html) | Prostatic Adenocarcinoma | 2 | 6(3+3) | n/a | n/a | M | 80 |
| [T3-031](http://www.proteinbiotechnologies.com/products/prostate/t3_031_human_prostate_tissue_lysate.html) | Prostatic Adenocarcinoma | 2 | 7(4+3 | n/a | n/a | M | 71 |
| [T3-032](http://www.proteinbiotechnologies.com/products/prostate/t3_032_human_prostate_tissue_lysate.html) | Prostatic Adenocarcinoma | 1 | 5(3+2) | III | T3bN0M0 | M | 65 |
| [T3-033](http://www.proteinbiotechnologies.com/products/prostate/t3_033_human_prostate_tissue_lysate.html) | Prostatic Adenocarcinoma | 1 | 4(2+2) | n/a | n/a | M | 69 |
| [T3-034](http://www.proteinbiotechnologies.com/products/prostate/t3_034_human_prostate_tissue_lysate.html) | Prostatic Adenocarcinoma | 2 | 7(4+3) | n/a | n/a | M | 78 |
| [T3-035](http://www.proteinbiotechnologies.com/products/prostate/t3_035_human_prostate_tissue_lysate.html) | Prostatic Adenocarcinoma | 2 | 7(4+3) | n/a | n/a | M | 43 |
| [T3-036](http://www.proteinbiotechnologies.com/products/prostate/t3_036_human_prostate_tissue_lysate.html) | Prostatic Adenocarcinoma | 2 | 5(2+3) | II | T2N0M0 | M | 62 |
| [T3-037](http://www.proteinbiotechnologies.com/products/prostate/t3_037_human_prostate_tissue_lysate.html) | Prostatic Adenocarcinoma | 3 | 9(4+5) | III | T3bNxMx | M | 64 |
| [T3-038](http://www.proteinbiotechnologies.com/products/prostate/t3_038_human_prostate_tissue_lysate.html) | Prostatic Adenocarcinoma | 1 | 6(3+3) | n/a | n/a | M | 74 |
| [T3-039](http://www.proteinbiotechnologies.com/products/prostate/t3_039_human_prostate_tissue_lysate.html) | Prostatic Adenocarcinoma | 2 | 8(5+3) | n/a | n/a | M | 47 |
| [T3-040](http://www.proteinbiotechnologies.com/products/prostate/t3_040_human_prostate_tissue_lysate.html) | Prostatic Adenocarcinoma | 3 | 9(4+5) | n/a | n/a | M | 71 |
| [T3-041](http://www.proteinbiotechnologies.com/products/prostate/t3_041_human_prostate_tissue_lysate.html) | Prostatic Adenocarcinoma | 2 | 7(4+3) | n/a | n/a | M | 87 |
| [T3-042](http://www.proteinbiotechnologies.com/products/prostate/t3_042_human_prostate_tissue_lysate.html) | Prostatic Adenocarcinoma | 2 | 8(5+3) | n/a | n/a | M | 71 |

Statistical analysis of data in Figure 2A

Statistical analysis of data in Figure 2D

Statistical analysis of data in Figure 3
